# Methane Emission of Italian Mediterranean Buffaloes Measured Using a Laser Detector During a Lactation Cycle

**DOI:** 10.3390/ani14243652

**Published:** 2024-12-18

**Authors:** David Meo Zilio, Miriam Iacurto, Francesco Cenci, Roberto Steri

**Affiliations:** 1 Consiglio per la Ricerca in Agricoltura e L’Analisi dell’Economia Agraria, Centro di Ricerca per la Zootecnia e L’acquacoltura, Monterotondo, 00015 Rome, Italy; miriam.iacurto@crea.gov.it (M.I.); roberto.steri@crea.gov.it (R.S.); 2 Associazione Allevatori dell’Umbria e delle Marche, Corciano, 06073 Perugia, Italy; f.cenciagronomo@gmail.com

**Keywords:** greenhouse gas, portable detector, sustainable, selection, milking

## Abstract

In Italy, the number of farmed dairy buffaloes rose up to approximately 436,000 heads in 2023 (+22% in the last 15 years), a fourfold increase compared to the 1980s, due to the growing market interest in mozzarella cheese. The increased demand for mozzarella cheese, in turn, requires higher production, which can result in increased methane emission from the sector. Therefore, it is necessary to establish mitigation and selection schemes for low-emission strategies. The current study aimed to highlight sources of variation in methane emission from lactating Italian Mediterranean buffaloes measured using a laser methane detector in order to identify practical and methodological aspects to consider when designing experiments focused on methane emission evaluation. Methane (CH_4_), exhaled from 60 cows, was recorded twice a day during milking, over two weeks per month for at least three months throughout a whole lactation cycle. The animal (individual), days in milk, parity, month, operator, milking entry order, and milking session effects were significant for methane emission (*p* < 0.0001). Our results showed that laser methane detector may be used as a rapid tool for methane emission studies and highlighted which factors can account for individual measures. This instrument is easy to use, fast, versatile, and not too expensive. These characteristics make it suitable for large-scale herd screening and monitoring.

## Simple Summary

There are about 200 million buffalo heads worldwide, representing a valuable source of food and, in many developing countries, also animal draft power. In Italy, buffalo farming produces a top-quality Italian food, namely “mozzarella di bufala”, which is exported globally and plays a major role in the sector’s economy. Being the livestock sector under severe criticism for its contribution to environmental impact of human activities, it is imperative to control greenhouse gas (GHG) emissions. Indeed, ruminants are gas emitters by nature owing to their digestion physiology. Understanding the emission pattern and amounts, at an individual level, as well as the factors involved in their modulation, is pivotal to achieving the objective of the reducing GHG emission from livestock farming. A laser detector, specific for methane (LMD), may be used to perform rapid on-farm measurements but physiological, environmental, and technical factors may affect the results. This study represents a first assessment of factors influencing methane emissions in lactating Italian Mediterranean buffaloes measured using a laser detector. Our results showed that individual, climate, and management factors should be considered when setting up protocols for LMD measurements.

## 1. Introduction

In Italy, Mediterranean buffaloes are mainly farmed for milk production, which is used for dairy processing into “mozzarella” cheese. The growing interest in “mozzarella” has led to an increase in the number of farmed dairy buffaloes, which, in Italy, rose up to approximately 436.000 heads (+22% in the last 15 years) in 2023 [1], a fourfold increase compared to the 1980s [2,3]. It is well known that ruminants emit methane (CH_4_), a potent greenhouse gas, as a product of the normal physiological enteric fermentations during the digestive process. CH_4_ is indeed continuously breathed and eructed while hindgut emission is negligible (less than 10%) [4]. Over the last few decades, several techniques have been developed for measuring ruminants’ methane emission based on breath analysis. The gold standard technique for in vivo individual methane emission is represented by respiration chamber (RC) [5], which consists of a strictly controlled chamber with sensors and flow meters, air conditioning, an alarm system, feeding trays, manure carts, and other equipment. Other commonly used techniques include the GreenFeed^®^ (GF), which is a type of transportable feeder that detects the gas emitted by the animal when its head is inside, and the sulfur exafluoride (SF_6_) technique [6]. However, those methods are costly and time- and labor- intensive and may be prone to operational issues that impact on efficiency and reliability. For example, RC and GF, besides the high purchase, installation, and running costs, have a limited operational capacity (the RC is individual and normally a subject is kept for 2–4 days, while the GF can manage up to around 20 animals over seven-day recordings). This last issue reduces the suitability of a method for genetic evaluations [5]. In addition, operating conditions are very different from the normal life of the animal (RC) and there is a need for specific training of animals and operators (GF) and for routine calibration. The SF_6_ method includes a direct procedure on the animal with the insertion of a permeation tube in the rumen and a series of sampling (for five sequential days over 24-h intervals), calibrations, and gas chromatography analysis [6,7]. Therefore, there is a strong motivation to find cheaper, faster, non-invasive and more versatile methods to measure individual CH_4_ emissions. Among these are the sniffers, usually coupled with an automatic milking system (AMS), used in the dairy cattle for measuring CH_4_ and for their potential application in genetic improvement [8]. The laser methane detector (LMD) may represent another suitable option [9], independent of the presence of an AMS, which is rarely used in buffalo farms [10,11].

An LMD is a handheld auto calibrating [12] and portable tool developed for the detection of gas leaks from a distance in gas pipelines, landfills, and other areas. In the last decade, its use in detecting livestock methane emission has been widely reported and has included different species such as dairy cattle [13,14,15,16,17,18,19], beef cattle [20], sheep [20,21], and goats [22,23], while there are only a few preliminary studies on buffaloes [24,25]. The LMD uses a high selectivity infrared absorption spectroscopy method to detect the CH_4_ concentration in the breath of animals [24,25]. A visible pointing system helps to direct the measuring laser to the desired target. The backscattered laser beam is converted by the transceiver into the CH_4_ concentration between the detector and the target, expressed as part per million per meter (ppm*m).

Ref. [19] demonstrated that LMD can be used in fast phenotyping of cows for CH_4_ under some circumstances. As shown by [18,26], the LMD technique may indeed be able to detect differences in CH_4_ emission caused, for example, by a feeding event. Ref. [15] reported a positive relationship between LMD and RC data and concluded that the LMD could rank cows for CH_4_ production. When compared with other methods, such as the GF and infrared sensors installed in an automatic milking system, the LMD method ranked cows similarly with respect to their CH_4_ emission [19]. Nevertheless, ref. [26] did not find any correlation between LMD and RC measurements, confirming what was stated by [20]. The LMD operates directly on breath CH_4_ concentration, which may depend on different parameters. Therefore, it is likely subject to high variance and uncertainty compared with quantitative direct measurements (e.g., RC). Methods based on concentration are less precise and accurate than flux methods (e.g., GF, SF_6_) but they are viable for large-scale measurement. Further development is needed to increase the accuracy and precision of concentration methods [5]. Additionally, the accuracy can be affected by the proximity of other individuals or methane sources, the distance to the target, the angle of the laser beam, and the distance to feeding [26]. All these factors may be difficult to control, adding some uncertainty. As for sensitivity, ref. [15] evaluated the ability of the LMD to detect high CH_4_ levels (above the third quartile of all values) similarly to the RC sensor and reported a sensitivity of 95% and 94% and a specificity of 97% and 79% for cows and sheep, respectively.

Moreover, due to its relatively recent use and the fact that this kind of device was designed for practical use on field (methane leak detection), it is important to establish a robust and standard protocol to effectively use the LMD on animals [16].

Physiological, management, and environmental factors, including season, parity, days in milk (DMI), climatological parameters inside and outside facilities, and others, can influence CH_4_ emissions. These factors may also interfere with instrumental responses. Therefore, it is important to study these effects along with other sources of variation, uncertainty, or bias due to experimental, human, and procedural factors.

Buffalo is supposed to be a lower emitter when compared to cattle [27] and given the size of buffalo farming industry in Italy, it is essential to increase the knowledge about the CH_4_ emission physiology and yield in this species and to refine its management for a more sustainable livestock system. Unfortunately, to the authors’ knowledge, there are only a few studies specific to buffalo, with most of the literature focusing on cattle and, to some extent, sheep. Additionally, most of the research on buffalo consists of estimates or predictions based on the statistical model [28] or on equation [29,30,31] from International Panel on Climate Change (IPCC). Ref. [32] used the SF_6_ technique while only [24] measured individual emission using LMD. Therefore, the aim of the present study was to (1) to explore practical and methodological aspects related to the LMD methane assessment; (2) to provide evidence-based on-farm collected data on the CH_4_ emission of lactating buffalo; and (3) to evaluate the main environmental, management, and individual effects on CH_4_ emissions of buffaloes during milking as well as the individual variability of this phenotype measured with LMD.

## 2. Materials and Methods

### 2.1. Animals, Diet, and Facilities

The study was conducted at the dairy farm of the “CREA, Research Center for Animal Production and Aquaculture” (Monterotondo, Rome, Italy, 42°04′55.2″ N 12°37′48.3″ E) over a 10-month study from January to October 2022. At our latitude, buffalo calving season is concentrated in July to December (70 to 80% of births) as autumn is the natural mating season. However, in Italy, we used to de-seasonalize the herds by shifting the mating period to meet the highest market’s request for mozzarella cheese, which is in the spring/summer period [33]. This way, births occur all year long starting from January.

To have a representative number of animals at different days in milk (DIM), all animals being in lactation within 1 to 270 DIM in that period (January to October) were included in the study and subjected to two consecutive weeks of measures per month for at least three months (animals exceeding 180 DIM at the beginning of the survey period and animal giving birth after 31 august 2022 were excluded from the study). The Temperature Humidity index (THI) and daylength of the considered period is reported in Appendix B. In total, 60 healthy pluriparous lactating Italian Mediterranean buffaloes were enrolled (liveweight 732.20 kg ± 50.64 kg; body condition score 7.38 ± 0.57). A Total Mixed Ration (TMR) was administered once a day (10.30 a.m.). Water was provided “ad libitum” in dedicated drinking troughs. No procedures were performed directly on animals. During the study, samples of the TMR were collected and analyzed for chemical composition with respect to the dry matter content (DM) and ash and crude protein (CP) following the AOAC methods [34]. Fiber fractions as neutral detergent fiber (NDF), acid detergent fiber (ADF), and lignin (ADL) were determined according to [35]. The results of the feed analysis are reported in Table 1. The animals were housed in pens in a free stall (north/south oriented) with feeding and resting areas on straw bedding that was renewed weekly and milked twice per day. The milking parlor was a herringbone model with 10 + 10 posts.

### 2.2. CH_4_ Emission Measurements

One-minute laser methane measurements were performed over two consequent weeks per month from January to October. Each animal was measured twice a day: during the morning (a.m.), 09:00 to 10:30, before feeding, and the afternoon (p.m.) from 17:30 to 19:00, milking sessions. Methane measurements were performed using a handheld auto calibrating (via an internal reference cell, https://www.fpi-inc.com/en/pages/productsDetail/productsDetail?id=100070 (accessed on 31 October 2024)) device RLGD-100 Remote Laser CH_4_ Detector (FPI, Hangzhou, China) and carried out by four trained operators, pointing the laser beam of the LMD at the nostrils of the buffaloes. The LMD concentration (ppm*m) of CH_4_ plume present in the laser beam pathway was recorded. The LMD technical specifications are reported in Appendix A. Although there is no consensus on the optimal measuring distance, distances between 1 and 3 m [12] have been used in the majority of studies regarding LMD, depending on the animals’ freedom of movement [8]. Ref. [36] demonstrated no differences within measures obtained at 2 or 3 m. The distance of 1 m guarantees a lower accumulation of background CH_4_ and a lower influence of environmental factors such as convective movements of the air or humidity but does not guarantee the complete exploration of the animal’s exhaled air during the respiratory cycle. On the other hand, measuring at a distance of 3 m could be susceptible to environmental factors. Therefore, we set a distance of 2 m (the maximum distance allowed by our facility) in order to minimize stress for animals and to keep operators safe. Measurements were taken on animals standing idle in the milking parlor post for 1‘each (one scan every 0.5 s) with the operator standing in front on the animal and pointing the laser beam directly on the muzzle. LMD measures between 1’ and 10’ long are reported in the literature [9]. In our study, we decided to adopt 1’ to sample as many animals as possible during the same event (milking) and to avoid stressing the animals by keeping them in the milking post for longer than usual and necessary. Additionally, this time slot is fully compatible with the milking routine, and it avoids limitations or interferences with operations. Based on this assumption, we extended the measures to twice a day for at least two weeks per month. This way, we obtained a large number of measurements consisting of 120 (two per second) scans per spot. In total, for each animal, we obtained around 3000-point measurements per month.

Data were then downloaded via Wi-Fi into a PC for storage and processing. The milking parlor has natural internal air recirculation, based on a chimney effect due to specific building features, that prevents the spent air accumulating at the animal level and interfering with measures. Anyway, before and after the milking session (i.e., before the first and after the last animal passed through in the milking line), the CH4 level of the ambient air was measured at a distance of two meters from the post by pointing the laser toward a panel in place of the animal, and no presence of gas was detected. The output of the LMD recording consists of a spectrum representing the values of CH_4_ emissions from a single animal with peaks representing breathes and burps. The intensity (goodness of the measure) and the concentration of methane emission (in terms of eructation peaks, breathing, and overall mean) were acquired for each sampling and used to perform the subsequent analysis.

CH_4_ measures were evaluated according to month, DIM class (each class consisting in 20 days of lactation), parity, milking order (number of the occupied post that stands for sequence of milking), milking session, rumination activity, laser operator, and mixer wagon operator, as reported in the following paragraph.

### 2.3. Data Process and Statistical Analysis

The final dataset contained records from 60 subjects with 669,556 observations and 84 spot measurements per animal on average. The LMD recorded the CH_4_ concentration every 0.5 s for a total of 120 records for each sampling and 240 records per animal/day. Measurements on the same animal were repeated for at least 12 days per month over at least three consecutive months.

**Figure 1:**
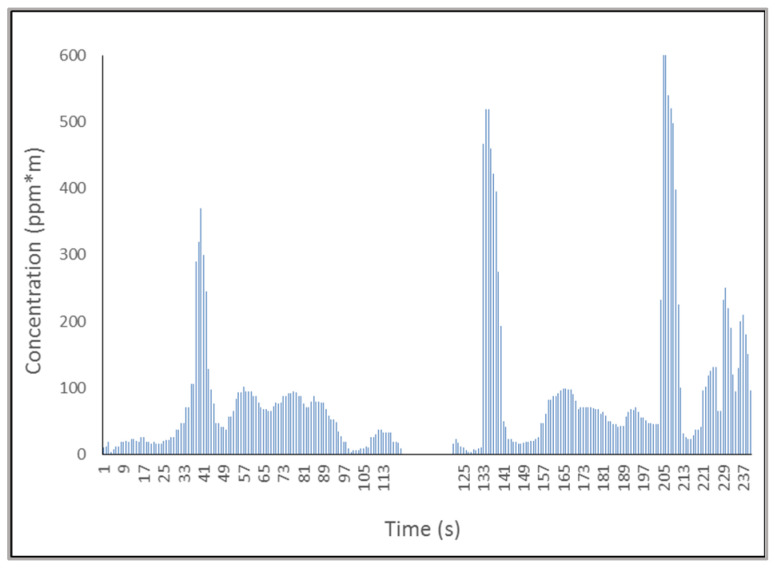
Daily exhalation profile during the morning (1–120 s) and afternoon (121–240 s) milking session.

The daily profile (i.e., a.m. + p.m.) of measurements of a single animal is reported in Figure 1. As widely reported in the literature for different ruminant species (i.e., cow, sheep), this profile presents specific values based on the respiratory cycle and well-defined eructation peaks.

Values higher than 2500 ppm*m were considered outliers due to errors in the reflectance of the laser beam [12], while the background concentration of CH_4_ was prudentially subtracted from the recorded raw measurements, considering the background level as the minimum measured value [37].

**Figure 2:**
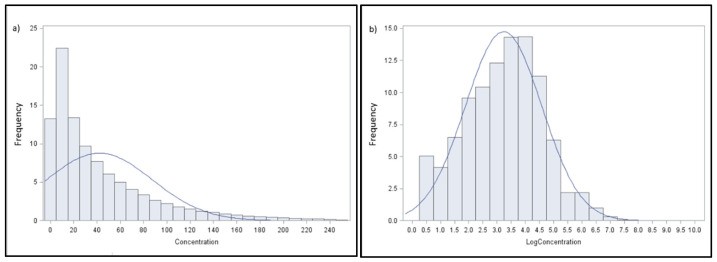
Distribution of CH_4_ emissions expressed as ppm*m (**a**) or as LogCH_4_ (**b**) *vs* normal distribution (blue line).

The distribution of the recorded variable is strongly asymmetric (Figure 2a); therefore, in order to obtain a normal distribution, the raw data were log-transformed [38] (Figure 2b).

After editing, the analyzed dataset contained 56 subjects and 455,079 records.

The CH_4_ phenotypic variation was analyzed with the following linear mixed model:

**y = M + DIM + P + MO + MS + R + LO + MWO + a + e**

where **y** was the LogCH_4_; **M** was the fixed effect of the month (10 levels: from January to October); **DIM** was the fixed effect of day in milk class (13 levels of 20 days of lactation each); **P** was the fixed effect of parity (5 levels: from the second to the sixth); **MO** was the fixed effect of milking order of entry into the milking parlor (20 levels); **MS** was the fixed effect of milking session (2 levels: morning, evening); **R** was the fixed effect of rumination activity (2 levels: yes, no); **LO** was the fixed effect laser operator (4 levels); **MWO** was the fixed effect mixer wagon operator (4 levels); **a** was the random animal effect; and **e** was the random residual effect.

The linear mixed model was solved using the MIXED procedure of SAS 9.4 software (SAS Institute Inc., Cary, NC, USA) [39] calling down the/solutions option for the random effect evaluation.

To further investigate the relationship between parity, milk, and CH_4_ production, the same model was fitted considering the interaction DIM*Parity and the distance from calving (day in milk) in days (270 levels) as a fixed effect.

## 3. Results

### 3.1. Methane Emission

The performed feed analyses are reported in the following table (Table 1).

The overall mean of CH_4_ emission, including breath and eructation, measured with the LMD, was 61.24 ± 114.2 ppm*m or 3.23 ± 1.35 when log-transformed. Except for rumination activity, each considered main factor significantly influences CH_4_ emission (Table 2).

#### 3.1.1. Animal-Based Factors

**Figure 3:**
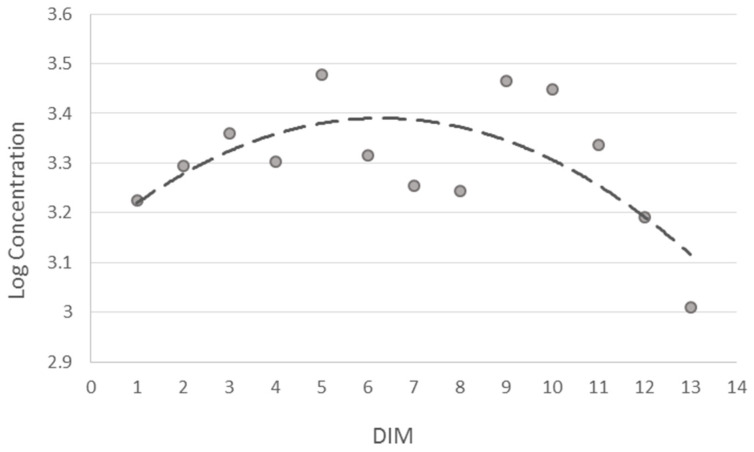
Estimated least means squares for DIM class (filled dots) and second-degree polynomial fit (dashed line).

**Figure 4:**
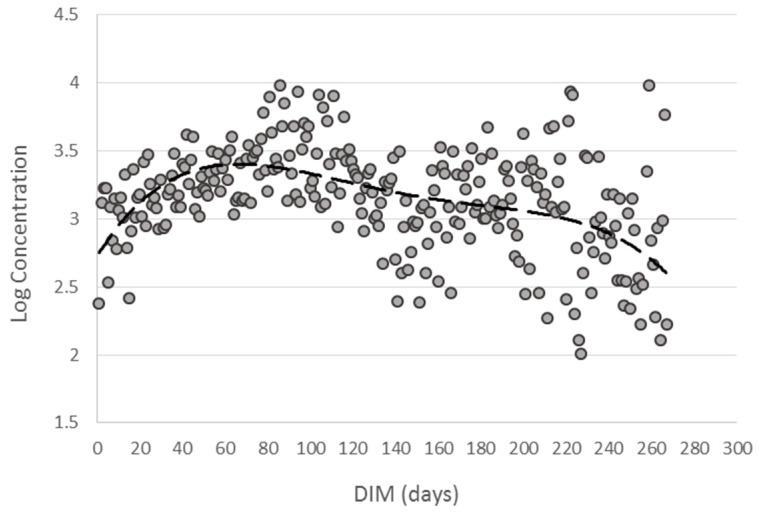
Fourth degree polynomial (dashed line) fitted on least square means (filled dots) for the fixed factor DIM.

Among the factors supposed to influence emissions, only rumination did not show a significant effect (*p* = 0.3024). Distance from calving expressed as DIM affected methane measures (*p* < 0.0001). The trend of exhaled CH_4_ according to DIM is reported in Figure 3 and Figure 4. The estimated values tend to increase in the first phase of lactation and decrease subsequently. The estimated means for the DIM Class (Figure 3) and for the days of lactation (Figure 4) show considerable dispersion around the mean, but when modeled with a second or fourth degree polynomial, respectively, they show an increase in CH_4_ emissions in the first phase of lactation until the production peak, around 60 days, and a subsequent decrease in the second phase until the dry period.

**Figure 5:**
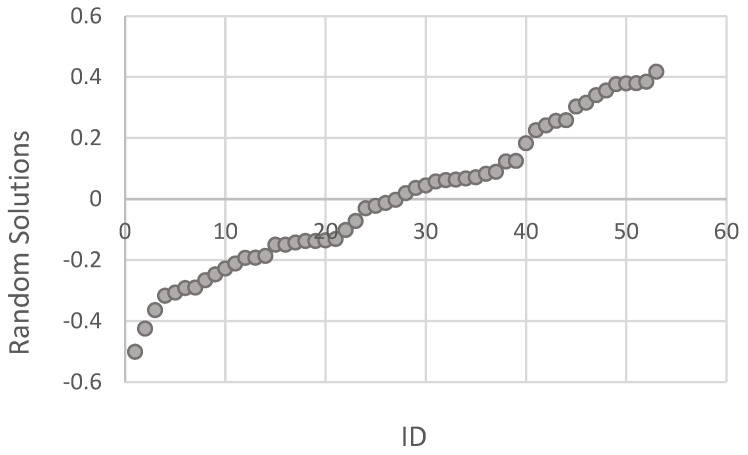
Distribution of solutions for the random effect ID in methane emission.

The random solutions for the animal effect considered in the model showed a strong and normally distributed individual variability (Figure 5). This result allows the ranking of the animals into high and low emitters.

**Figure 6:**
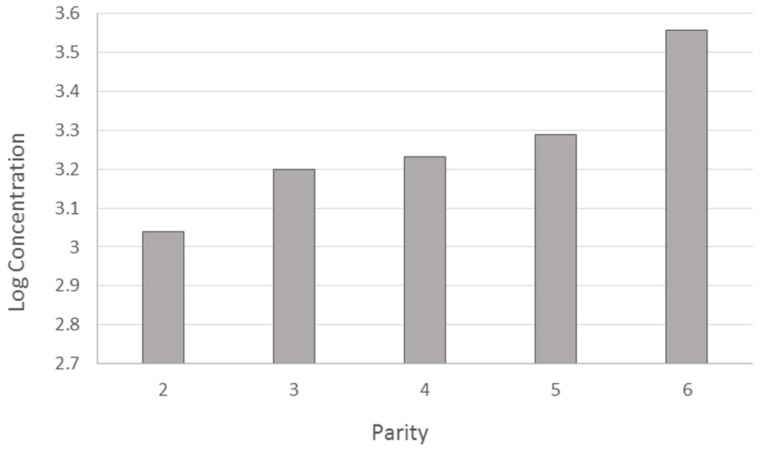
Estimated least means squares for parity on methane emission.

The CH_4_ concentration increases with parity from 2 to 6, with the buffaloes belonging to this last category being more of an emitter during milking (Figure 6). These results could be attributable both to the increase in weight (Table 3) and to the higher milk production with the increase in parity.

The mean liveweight (LW) within parity is reported in Table 3.

**Figure 7:**
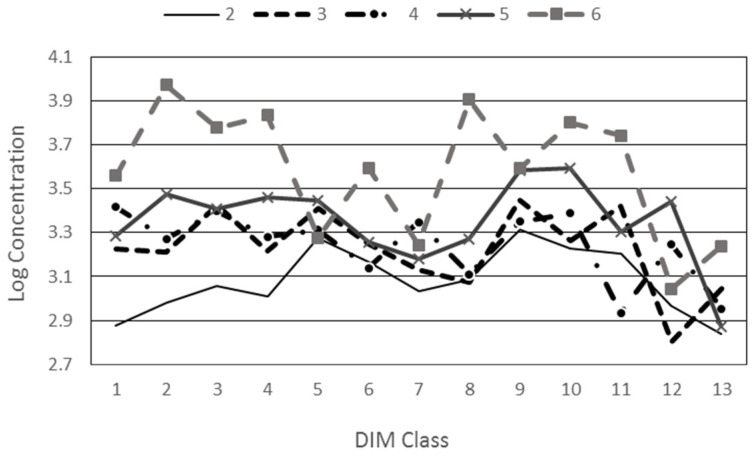
Parity × DIM interaction.

The interaction between parity and DIM is represented in Figure 7.

A clear separation can be found in the first four DIM classes, with emission levels increasing according to parity.

#### 3.1.2. Environment-Based Effects

**Figure 8:**
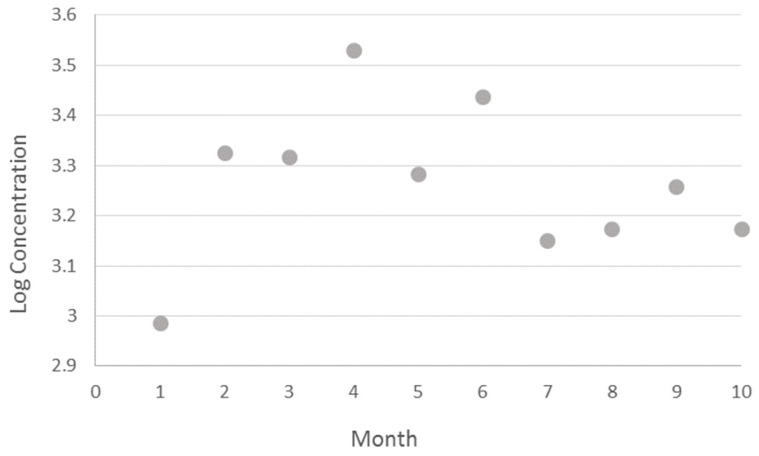
Monthly methane emission within the observed period.

**Figure 9:**
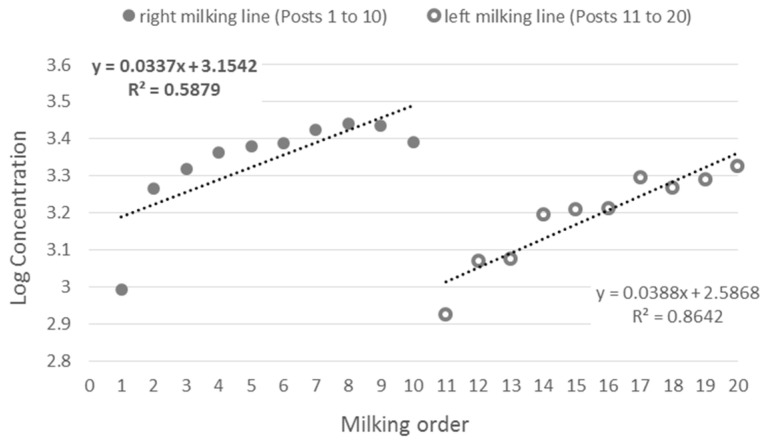
Variation in methane emission according to the milking post and milking line.

The highest methane emission was found in April, while the lowest was in January. The results are reported in Figure 8. Milking post also has influenced the measures (Figure 9). Posts from 1 to 10 were correlated to higher emissions than 11 to 20 (3.32 ± 0.03 vs. 3.18 ± 0.03, *p* < 0.0001).

#### 3.1.3. Management-Based Effects

Both mixer wagon and laser methane detector operators strongly influenced methane emission (*p* < 0.0001). Within this subsection, it is worth mentioning that the milking session (i.e., morning or evening) also had a highly statistically significant effect (*p* < 0.0001). The results are reported in Table 2.

## 4. Discussion

The aim of the study was to assess methane emissions in lactating Mediterranean buffaloes measured by using the LMD and propose an overview of their variations within the farming routine. Individual, environmental, and human-based factors have been considered. The LMD appears to be a suitable tool for rapid and low-cost monitoring of enteric CH_4_ and was confirmed to be versatile and reliable, allowing on-farm monitoring in a non-invasive way [24]. Only a few studies have dealt with buffalo methane emission so far, and more insight research is needed given the importance of this species, not only in Italy. Specifically regarding buffalo emission measured with an LMD, Ref. [24] used non-lactating animals and [25] pointed out the individual variability of the phenotype. Therefore, there is a lack of research on production, practical, methodological and other potentially confounding effects.

As for methodological aspects of LMD assessment, they still need to be standardized and generally accepted. Research [9] reports different times of data recording to provide reliable results for the quantitative estimation of methane production (e.g., kg/day or kg of CH_4_/kg of milk) with the duration of a single spot varying between 1’ and 10’. The LMD has been considered to cause low to medium alteration in animal behavior [5], with “medium” indicating some handling, training, or change in routine. We adopted a 1’spot measure to avoid a change in routine and animal handling or training. To minimize the duration of the measurements (1 min), we repeated the measure twice a day for two consecutive weeks during at least three months. This approach allowed us to gather a large number of spot data, increasing the statistical power by reducing the type 1 error. To minimize the effect of operations on animals and staff, we chose a measuring distance of two meters, the maximum allowed under our specific operational conditions. This distance is used within the Welfare Quality^®^ protocol [40] to perform the avoidance distance test, which involves approaching a cow immediately in front at a rate of 1 step/s and starting at 2 m [41] to evaluate fear response. Regardless of the absolute value of methane yield, the focus of this study was to assess how measurements were affected by different factors during the regular farming operations and whether animals could have been stratified in high and low emitter type. Emissions are known to be affected by the diet and in particular by fiber content [42,43,44]. In this study, a basal diet for milking buffaloes (Table 1) was used throughout the observed period, and no feeding treatments were applied.

Regarding the sources of variation within normal farming practice that have been considered, the distance from calving (DIM) was confirmed to be significant (*p* < 0.0001), as reported in Table 2. In general, the CH_4_ emission curve appears to be superimposable to the estimated lactation curve for the Mediterranean Buffalo [45]. A similar pattern was reported by [46] for dairy cattle. Several authors have reported increasing daily methane emissions during the first 10 weeks followed by a stationary phase [47,48].

A strong relation between DIM and CH_4_ was found on dairy cattle by [47], who stated that emissions increase up to 20 weeks of lactation and keep constant up to 50. The effect was explained by the same authors as being due to differences in the amount and quality of the diet. In our study, we used the same diet for all animals throughout the entire period. Our data showed a significant decrease corresponding to the higher phases of lactation (i.e., DIM class > 10) corresponding to the minimum yield [49,50], even though data are very dispersed around the mean (Figure 4). This result was not consistent with other studies on dairy cattle where no effect [51] or limited effect [47,48,49,50,51,52] (i.e., increase up to the first 20 weeks and persistence up to the fiftieth) were reported. The tendency to decrease might be related to environmental (i.e., hot season approaching, photoperiod) or physiological (i.e., milk yield, liveweight, BCS) effects.

Also, parity affected laser measurements. Parity is positively correlated with liveweight, so this effect may be a consequence of a higher feed intake and/or milk yield. Other studies have reported that the average milk yield increases with parity on buffaloes [50]. Milk yield is also related to DMI in cattle [53]. On the other hand, [54] found the older dairy cows are lower emitters, both in terms of g/min and g/kg of milk. The interaction between parity and DIM in terms of methane emission (Figure 7) could confirm that CH_4_ is influenced by production level, which in Mediterranean Buffalo increases up to the sixth lactation in terms of the total yield and peak production [32].

The effect of the month of sampling is evident in Figure 8 and it is consistent with findings from other investigators [24,25,26,27,28,29,30,31,32,33,34,35,36,37,38,39,40,41,42,43,44,45,46,47,48,49,50,51,52,53,54,55]. Those authors reported a lower emission in the hot season, due to heat stress, and consequent reduction in activity, DMI, gastrointestinal motility, and different hormonal balance. However, it is unclear why, within two well-differentiated levels of emission belonging to mild (higher emission) and hot period (lower emission), January segregates, showing the lowest value as reported in the figure. On the other hand, [56] did not confirm the seasonal effect, in terms of THI, on buffalo emission. Further in-depth study is necessary to explore the physiological, meteorological, and behavioral changes, according to seasonal variation, and their effect on methane emission. One challenging explanation could be the effect of photoperiod on methane emission, rather than the effect of the season “*per se*”, which might interfere with other seasonal factors like temperature or humidity. Ref. [57] reported that seasonal or photoperiodic-sensitive animals respond to altered day length with changes in physiology (growth, food intake, and reproductive status) and behavior to adapt to predictable yearly changes in the climate. Furthermore, an increase in milk yield was found by [58] on dairy cattle under long photoperiod condition, even though no higher DMI was found. Those results could also account for a change in CH_4_ production due to hormonal or other physiological factors. THI and daylength trends are shown in Appendix B. Therefore, a comprehensive study including production, reproduction, management, environmental, and endogenous factors would help to reach a consensus among scientists.

Among the other environmental influencing factors, milking order is most probably due to dominance in a hierarchical structure [59] and may be associated with milk yield, somatic cell count, and other parameters in dairy cow [60]. It remains to be clarified how this mechanism declines and why the last-entered cows within both the milking post lines are emitters more than the first. Eventually, regarding management, operators, of both laser methane detectors and mixer wagon, have significantly influenced the methane emission. Trained and skilled operators are fundamental when dealing with animals when using electronic or mechanical tools for precision application. A final aspect to be included in experimental design and analysis is fasting or eating and, if applicable, the distance from feeding. As shown in Table 2, the milking session had a strong influence on methane. This effect might be due to different levels of rumen fill reflecting various dry matter intake, ration composition, digestion, and rate of passage [61,62].

## 5. Conclusions

LMD has been used on different species and in different farming systems (i.e., extensive, semi-intensive, and intensive) with different protocols and different measurement specifications. Studies directly relating with buffalo are few and sectoral. To the authors’ knowledge, only two studies have been previously reported on this species, both suggesting that this method can be adopted to measure emissions of buffaloes under different scenarios. In this view, it is important to set up and to adjust rigorous protocols to purge the data of confounding effects and avoid bias in models and predictions. Indeed, methods based on concentration are supposed to be less precise than respiration chamber or other methods based on flux (for example GreenFeed^®^ or SF_6_). Our study highlights, for the first time, the main factors influencing CH_4_ emission, measured in a dairy buffalo farm, with this rapid and non-invasive system. Many issues should be taken into account. Individual (animal), environmental (climate), and managerial (farming) aspects must be regulated for reliable analyses. Trained, conscious, and motivated technical operators are necessary as suggested by the strong effect attributable to the operator. In conclusion, LMD is a rapid, inexpensive, and portable tool, suitable for use on a large number of animals. The laser detector applied to methane emission measurements can allow the individual discrimination of buffalo according to this phenotype, laying the foundations for setting up a genetic model, as has already occurred for cattle. In this context, further species specific (buffalo) studies are needed in order to develop and set up models based on precisely determined phenotypes and on robust predictions.

## Figures and Tables

**Table 1:** Dry matter (% as is) and chemical composition (dry matter basis) of the diet.

DM %	CP %	NDF %	ADF %	ADL %	Ash %
70.23 ± 1.19	15.69 ± 1.15	53.56 ± 4.25	26.71 ± 2.28	6.67 ± 1.40	6.67 ± 1.40

DM = dry matter; CP = crude protein; NDF = neutral detergent fiber; ADF = acid detergent fiber; ADL = acid detergent lignin.

**Table 2:** Main studied effects on individual methane emission.

Factor	DF Num *	DF Den *	F-Value	Pr > F
(DIM) DIM Class	13	4.50 × 10^5^	70.08	<0.0001
(P) Parity	4	4.50 × 10^5^	18.92	<0.0001
(M) Month	9	4.50 × 10^5^	86.81	<0.0001
(LO) Laser Operator	4	4.50 × 10^5^	204.15	<0.0001
(MO) Milking order	19	4.50 × 10^5^	59.45	<0.0001
(MS) Milking session	1	4.50 × 10^5^	1725.66	<0.0001
(R) Rumination	1	4.50 × 10^5^	2.59	0.3024
(MWO) Mixer wagon operator	3	4.50 × 10^5^	130.92	<0.0001

* DF Num = numerator’s degrees of freedom, * DF Den = denominator’s degrees of freedom.

**Table 3:** Mean liveweight (±s.d.) for different parity classes.

Parity	LW
2	719.3 ± 63.7
3	736.6 ± 60.3
4	740.7 ± 47.2
5	741.6 ± 35.9
6	746.4 ± 13.1

## Data Availability

The datasets analyzed during the current study are available from the corresponding author on reasonable request.

## Appendix A

Technical laser detector specifications provided by the manufacturer (FPI, user’s guide) are reported below.

**ITEM**	**DESCRIPTION**
Detection Method	Tunable Diode Laser Absorption Spectroscopy (TDLAS)
Detection Gas	Methane (CH_4_) or methane contained gases
Measurements Range	0~50,000 ppm*m
Detection Distance	0.5~30 m nominal. Actual detection distance may vary from objectives and conditions
Sensitivity	5 ppm*m at a distance from 0–10 m
Accuracy	±10% (100~1000) ppm*m
Response Time	0.1 s
Alarm Range	1–9999 ppm*m
Alarm Models	Audible tone, visual flash and concentration reported on the display
Selftest & Calibration	Built-in self-test and automatic calibration before startup
Laser Safety Class	In conformity with IEC60825-1.2007, a class 1 laser is equipped on this instrument as the detector and a class 2 or 3R laser used as the spotter
Explosion Proof	Ex ib IIA T3 Gb
Enclosure Protection	IP54
Operating Conditions	(−20~+50) °C, (30~95)%RH (non-condensing)
Storage Condition	(−30~+60) °C, <90%RH (non-condensing)
Power Supply	Rechargeable lithium-ion battery
Battery Duration	≥8 h at 25 °C under screen brightness level 3 and sound volume level 2
Battery Charging Time	≤4 h
Weight	<1.5 kg

## Appendix B

Daylength (blue line), maximum THI (red line), and minimum THI (green line) recorded during the studied period.
